# Wilde’s worlds: Sir William Wilde in Victorian Ireland

**DOI:** 10.1007/s11845-016-1428-4

**Published:** 2016-03-18

**Authors:** J. McGeachie

**Affiliations:** Centre for the History of Medicine in Ireland, School of English and History, Ulster University, Shore Road, Newtwnabbey, Co. Antrim BT37 OQB Northern Ireland, UK

**Keywords:** Parallels with Darwin’s early career, Network of enterprises, Centres of calculation, Savant Dublin, Victorian elite medicine, Irish complexities, National catastrophe, medical deliverance from, Irish tradition of scientific medicine

## Abstract

**Introduction:**

Other contributors to this collection have evoked the disparate worlds inhabited by Sir William Wilde.

**Aims:**

To provide an overall assessment of his career.

**Materials and methods:**

Looking at the historical conditions that made possible such a career spanning such disparate worlds. Deploying methodologies developed by historians of medicine and sociologists of science, the article brings together Wilde the nineteenth century clinician and Dublin man of science, the Wilde of the Census and of the west of Ireland, William Wilde Victorian medical man and Wilde the Irish medical man—the historian of Irish medical traditions and the biographer of Irish medical men, and William Wilde as an Irish Victorian.

**Conclusions:**

A variety of close British Isles parallels can be drawn between Wilde and his cohort in the medical elite of Dublin and their clinical peers in Edinburgh and London both in terms of clinical practice and self-presentation and in terms of the social and political challenges facing their respective ancient regime hegemonies in an age of democratic radicalisation. The shared ideological interests of Wilde and his cohort, however, were also challenged by the socio-political particularities and complexities of Ireland during the first half of the nineteenth century culminating in the catastrophe of the Great Famine. William Wilde saw the practice of scientific medicine as offering a means of deliverance from historical catastrophe for Irish society and invoked a specifically Irish scientific and medical tradition going back to the engagement with the condition of Ireland by enlightened medical men in the seventeenth and eighteenth centuries.

In looking at the roots of the disparate involvements that characterised Wilde as a Dublin savant, a man of science I draw on the work of the Cambridge historian of science Martin Rudwick for the concept of a ‘network of enterprises’; and also on that of the French social theorist Bruno Latour for his concept of ‘centres of calculation’.

As a medical student, Wilde was mentored by two of his teachers, the physicians Robert Graves and William Stokes. It was through their patronage that immediately after the completion of that training he went on to acquire a ‘network of enterprises’ comparable to that which Martin Rudwick has shown the young Charles Darwin acquiring whilst living in London after returning from the * Beagle* voyage [1].

Through the patronage network of *his* Cambridge mentor, the botanist JB Henslow, Darwin had been appointed resident naturalist to HMS Beagle after he graduated. The writing up and publishing in 1839 of the observations and researches Darwin had gathered during the ship’s extended voyage of scientific exploration between 1832 and 1836 established his credentials as a savant in the institutions of the British scientific establishment. It also laid the foundations for the wide-ranging but interlocking research trajectories—the ‘network of enterprises’—through which Darwin’s work on species was put together.

Likewise, Wilde’s Dublin mentors, Graves and Stokes, ensured that the completion of his medical apprenticeship in 1834 was followed by a period of voyaging roughly contemporary to that of Darwin and which similarly laid the foundations of Wilde’s own ‘network of enterprises’ as a Dublin savant. Writing up his researches and observations whilst travelling on the steam yacht *Crusader* as medical attendant to a wealthy invalid [2, 3], led to the 1840 publication of *Narrative of a voyage to Madeira, Tenerife and along the shores of the Mediterranean* after Wilde’s return to Dublin [4]. This became an instant best-seller, becoming known as *Wilde’s Voyage*, just as the lengthy full title of Darwin’s 1839 *Journal of Researches into the geology and natural history of those countries visited by HMS Beagle under the command of Captain Fitzroy, RN, from 1832 to 1836* became popularly abbreviated to *The voyage of the Beagle* [5]. *Wilde’s Voyage* was a two-volume account of the natural history and topography, climate and medical provision to be found in the places visited by the Crusader. It also included extensive reflections by Wilde on contemporary debates about the Levantine origins of the Celt—Norman Vance’s ‘Celts and Carthaginians’ [6]—prompted by his visits to the extensive ruins of the ancient civilizations of the eastern Mediterranean.

The *Crusader* had functioned for Wilde as the Beagle had for Darwin. This was as the research base for a voyage of scientific exploration, what Bruno Latour has called a ‘centre of calculation’ from which scientific and geographical information could be disseminated to imperial centres [7]. And although Wilde’s ship, the *Crusader* was a privately owned vessel, not a ship of the Royal Navy like the *Beagle*, and Wilde was privately employed as a medical attendant to a private individual, not to a captain of the Royal Navy as was Darwin, the *Crusader’s* voyage, like that of the Beagle, was through seas controlled in this post-Napoleonic era by the Royal Navy; its provisioning and postal communications likewise relying on outposts of British imperial power.

The book which came out of the voyage displayed the field work of an articulate naturalist in exotic regions. It also represented a contribution to the burgeoning genre of medical geography and travel writing, a genre pioneered by the Scottish physician, Sir James Clark, and which catered for the desires of wealthy Victorians for heathy convalescent locations, with the added bonus of landscapes of scholarly interest. Furthermore, Wilde’s book could be read as a contribution to the musings, both ethnological and antiquarian, of Irish and British celticists. A marker as to the wider, imperial readership for whom the book would have been intended is Wilde’s frequent self-designation as ‘English’.

In a specifically Irish context, *Wilde’s Voyage* provided its author with a gateway into the *savant* institutions of Dublin—the Royal Irish Academy, the Royal Dublin Society, the Royal Colleges of Physicians and Surgeons and Trinity College—in a way comparable to what the *Beagle* voyage and resultant researches were doing for Darwin’s position within London science at the same time. Indeed, a further Wilde-Darwin parallel can be drawn here, with Wilde’s period living on Westland Row before moving to the more spacious and prestigious address of number 1, Merrion Square, and Darwin initially living on Gower Street where the University College London anatomy building stands today before adopting the life of a country gentleman in Down House in Kent.

Furthermore, the exploration of the Levant was as crucial for Wilde’s subsequent medical trajectory as travel to South America and the Galapagos Islands were for that of Darwin within the bio-medical sciences. In Egypt he was able to observe at first hand the specialist facilities established by the modernising pasha, Mohammed Ali for the treatment of the infectious ophthalmic trachoma which had been brought to western Europe, including Ireland, as a consequence of Napoleon’s invasion of Egypt. This experience gave Wilde what became a lifelong interest in diseases of the eye, while the income generated by his book facilitated the development of that interest in London at Moorfields.

That income also helped lay the roots of his other medical specialism by permitting further travel to Vienna and Berlin. At the Allegemeines Krankenhaus in Vienna he encountered at first hand the beginnings of the systematic study and practice of otology. Vienna also gave him the opportunity to research a second book, the 1843 *Austria:Its literary, scientific and medical institutions* [8], for which he had to gain the statistical skills in the burgeoning field of public health and demography that facilitated his appointment as commissioner to the 1841 Irish Census. In Berlin thereafter, the ethnological interests acquired during his voyage were stimulated by the introductions to Alexander von Humboldt and the Berlin Anthropological Society given to him by Maria Edgeworth. All of this further enhanced his reputation in *savant* Dublin.

The network of enterprises that Wilde had acquired thus provided him with the stage on which he could combine the roles of clinician, savant, polymath and public intellectual. Although the area of medical practice in which he achieved such prominence was highly specialised compared to those of his mentors, the surgeon Abraham Colles, the physicians Graves and Stokes, the extent of his public and scholarly hinterland was remarkable, even by the standards of his day.

That hinterland encompassed the Irish census, the Ordnance Survey and the statistical and public health movements, on the one hand, and the periodical journalism of the *Dublin University Magazine* together with the naturalists, ethnologists, antiquarians and archaeologists of the Royal Irish Academy, on the other [9]. The catalogue of antiquities he compiled for the Academy was the first of its kind in Europe. Knighted in 1864 for ‘Services to the Irish Census’, Wilde was awarded the Royal Irish Academy’s Cunningham Prize during the same year in honour of his work as an antiquarian.

Upon his return from London and central Europe to establish his specialised practice in Dublin, Wilde became a fully fledged member of the generational medical cohort around Graves and Stokes in the nineteenth-century heyday of Dublin medicine. Like other ambitious Victorian clinicians, Wilde sought to further his career and influence through a period of medical editorship, seeking to enhance the prestige of the foremost Irish medical journal of his day by turning it into a quarterly title. Again, like many other Victorian medical men he supplemented his income whilst establishing himself in his practice by working as a medical adviser to an insurance company. And alongside of all of this was his demographic work for the Census and the associated publications.

The Graves-Stokes- Wilde cohort or axis within Dublin medicine, like those of their clinical peers in Edinburgh and London, were medical modernisers and reformers with international reputations. Like many of those Edinburgh and London peers, and as Wilde himself was later to do, they too had also taken advantage of the enduring European peace after the Napoleonic Wars, to enhance their medical educations overseas—medical grand tours.

Wilde’s cohort also shared with their clinical peers on the other island a commitment to a gentlemanly rather than professional model for the practice of elite medicine. They regarded themselves as medical men [10], men of science [11], and in many cases men of letters [12] rather than professional clinicians and scientists.

Indeed, most historians of nineteenth-century medicine and science now question the extent to which there was a decisive shift towards professionalization during the nineteenth century and the extent to which the medicine practiced during that period was scientific in the way that we understand that term today. What does seem clear is that the *representation* of the medicine practiced by these groups as being a science-based activity was central to their self-presentation of themselves as men of science. In other words, what they called scientific is not necessarily what we would call scientific. And frock-coats rather than white coats remained the norm in Merrion Square as in Harley Street. Laboratory-based medicine was still largely in the future during Wilde’s lifetime.

Calling what you did scientific was in fact an important rhetorical resource in what Frank Miller Turner has called the contestations of cultural authority, the culture wars that were a central feature of early-Victorian intellectual culture where ‘various groups of Victorian intellectuals… (attempted) to propose themselves as new authorities, or to resist the challenge of newcomers and to preserve earlier ideas and values in novel guises and institutional arrangements’ [13].

Wilde’s medical cohort belonged to the second of Turner’s categories. Its members were members of a largely Anglican Irish elite during the period after the Union but before the 1868 Disestablishment of the Church of Ireland. They were closely associated with Trinity College Dublin, an institution founded on the Oxford-Cambridge, Anglican collegiate model. The *ancien regime* hegemony they enjoyed was subject to similar challenges to those understood by the Coleridgean conservatives who led the London medical elite at that time [14].

But where the primary challenge posed to the London elite was perceived to be French materialist theories of the body inspired by Lamarck’s evolutionism and adopted in the private medical schools of the imperial metropolis, the challenges perceived by Wilde and his colleagues were compounded by specifically Irish political complexities and ambiguities. Stokes, Graves and Wilde were children of counter-revolution, members of a generation growing up during the uneasy decades that followed the Union after what was remembered—when not half-suppressed as a memory—as the catastrophe of 1798. These were the decades of the Ribbonmen, the Tithe War and acute agrarian unrest, of O’Connell and the political rebirthing of Catholic Ireland; decades experienced in terms of increasingly apocalyptic foreboding in the religious and political world of Wilde in particular, as the son of a protestant country doctor in Roscommon accompanying his father on his rural rounds.

Those forebodings are gothicly evoked in the passage in Wilde’s 1852 book, *Irish popular superstitions* when ‘ribbonism…sank deep and spread wide throughout the peasant and small-farmer class of the hitherto peaceful barony of Ballintober’ [15]. A young local is suborned into joining a nocturnal expedition of ribbonmen and killed during a police ambush; his corpse subsequently paraded on a cart through the streets of Roscommon town, Strokestown and Wilde’s native Castlerea bearing a sign pinned to its chest proclaiming ‘I am a ribbonmen,’ while others accused of ribbonism are flogged behind the carts which follow.

The counter-revolutionary terror of the 1820s and 30s, when a woman-hangman known as Lady Betty presided over Roscommon jail was followed by the Famine and its catastrophic effect on the west of Ireland with which Wilde as antiquarian, archaeologist and celticist was so obsessed. The interests in antiquarianism, in archaeology and folklore he had acquired whilst accompanying his father on his rounds in rural Connaught, however, were politically more complex than the establishment celticism he shared with other Dublin savants. He felt a sympathetic affinity with the nationalist predilections of many of the topographers and Irish language scholars working on the Ordnance Survey [16] while through his wife, *Speranza* [17], he was also connected to the romantic nationalism of Young Ireland [18]. In his final years he gave his support to Isaac Butt’s campaign for Irish home government.

By virtue of the ideological interests he shared with his cohort and through his work as a census-commissioner, Wilde himself was an actor in the post-Union project of transforming Irish society and assimilating it into the polity of the United Kingdom of Britain and Ireland. But the catastrophe of the Famine subverted the assimilation of Irish actuality into imperial quantification in the 1851 Census *Report on the table of deaths* [19]. Over the course of writing the 1851 census report, Wilde’s authorial voice moved from being that of a government-appointed exponent of one of the key legacies in Ireland of the Enlightenment state—the tabulating of public health—to that of the narrator of a tragedy, the massive human, social and cultural catastrophe of the Famine.

For Wilde, however, scientifically informed medical practice provided a means of deliverance from both the immediate catastrophe of the Famine and the long history of natural and political catastrophe that had preceded it. This is why the assured tone of scientific modernity of his 1853 textbook *Practical observations on aural surgery and treatment of diseases of the ear* [20] contrasts so markedly with the tone of romantic tragedy, the emphasis on the dark determinants of catastrophe culminating in the Famine described in the 1852 *Irish popular superstitions* and in the 1851 census report which he was also writing in the early 1850s.

This sense of a medical science that can bring enlightened improvement to Irish society is also found in the biographical essays on Irish medical men from the late seventeenth-century into his own time that Wilde initiated in the *Dublin University Magazine* with his 1841 four-part essay on Sir Thomas Molyneaux in a series of ‘Lives of illustrious Irishmen’, and continued in the *Dublin Quarterly Journal of Medical Science* with a series of extensive studies of ‘Illustrious Irish physicians and surgeons’ written by Wilde and others.

That legacy is also given a specific institutional history with the extensive ‘Notices of the medical and philosophical societies of Dublin’ with which Wilde in 1845 began the first issue of the *Dublin Quarterly Journal of Medical Science* under his editorship and in his 1846 ‘Memoir of the Dublin Philosophical Society’ [21]. In these writings, Wilde was cumulatively attempting to construct an Enlightenment narrative of scientific rationality for himself and his contemporaries which went back to the engagement with the condition of Ireland by Molyneaux, Sir William Petty and their fellow-members of the Dublin Philosophical Society in the 1680s [22].

Taken together, these essays link Wilde’s contemporaries– Sir Henry Marsh, Robert Graves, Sir Robert Kane and John Philpot Curran with the Enlightenment luminaries of Irish medicine—Molyneaux, Richard Steevens, Sylvester O’Halloran and Bartholemew Mosse. But while Enlightenment values continue to be invoked throughout this series, new notes come in, those of romantic genius with the 1842 Graves essay and the romanticist cult of martyrdom in that of 1847 on Curran, himself a victim of the Famine, dying as a result of ministering to his patients, in what Wilde described as the ‘feversheds of Kilmainham’.

Thus, in terms of any overall assessment of Sir William Wilde, we see, on the one hand, the British Isles typicality of someone who trained and practiced as a medical man at a time when medical men were re-defining themselves as men of science, and on the other, the historical peculiarity within these islands of Irish political experience and its wider ramifications. Someone who saw science and medicine as offering in the wake of the political autonomy lost with the Union and the national tragedy brought by the Famine, some possibility of redemption from Irish history and a better accommodation with the wider imperial entity brought by that Union.

## References

[1] Rudwick M (1982). Charles Darwin in London: the integration of public and private science. Isis..

[2] McGeachie J (2004) Wilde, Sir William Robert Wills, 1815–1876. In: Oxford dictionary of national biography. Oxford University Press, Oxford http://oxforddnb.com/view/article/29403. Accessed 7 July 2015

[3] Wilson TG (1942). Victorian doctor; being the life of Sir William Wilde.

[4] Wilde WR (1840) Narrative of a voyage to Madeira, Tenerife and along the shores of the Mediterranean, including a visit to Algiers, Egypt, Palestine, Tyre, Rhodes, Temessus, Cyprus and Greece: With observations on the present state and prospects of Egypt and Palestine and on the climate, natural history, antiquities etc., of the countries visited. Curry Dublin. A second edition, enlarged and revised was published in 1844

[5] Darwin C (1839) Journal of researches into the geology and natural history of the various countries visited by H. M. S. Beagle under the command of Captain Fitzroy, R. N., from 1832 to 1836. H. Colburn, London

[6] Vance N (1980). Celts, Carthaginians and constitutions: anglo-Irish literary relations, 1780–1820. Ir Hist Stud.

[7] Latour B (1987). Science in action: How to follow scientist and engineers through society.

[8] Wilde WR (1843). Austria: Its literary, scientific and medical institutions: With notes upon the present state of science and a guide to the hospitals and sanitary institutions of Vienna.

[9] McGeachie J (1999) Normal developments in an abnormal place: Sir William Wilde and the Irish school of medicine. In: Jones J, Malcolm E (eds) Medicine, disease and the state in Ireland, 1640–1940. Cork University Press, Cork, pp 86, 92–93

[10] Lawrence C (1985). Incommunicable Knowledge: science, technology and the clinical art in Britain, 1850–1914. J Contemp History.

[11] White P (2003). Making the man of science.

[12] McGeachie J, Cox C, Luddy M (2010). Science, politics and the Irish literary revival: Reassessing Dr. Sigerson as polymath and public intellectual. Cultures of care in Irish medical history, 1750–1970.

[13] Turner FM (1993) Contesting cultural authority: essays in Victorian intellectual life. Cambridge University Press, Cambridge

[14] Desmond A (1989). The politics of evolution: Morphology, medicine and reform in radical London.

[15] Wilde WR (1852, 1972) Irish popular superstitions. J. McGlashan, Dublin, 2nd edn. Shannon, Irish University Press

[16] Doherty GM (2006). The Irish ordnance survey: History, culture, memory.

[17] Melville J (1994). Mother of Oscar: the life of Jane Francesca Wilde.

[18] Quinn J (2015). Young Ireland and the writing of Irish history.

[19] Froggatt P (1965). Sir William Wilde and the 1851 Census of Ireland. Med Hist.

[20] Wilde WR (1853). Practical observations on aural surgery and treatment of diseases of the ear.

[21] Wilde WR (1844–1847) Memoir of the Dublin Philosophical Society of 1683. Proceedings of the Royal Irish Academy 3. 1846:160–176

[22] Coonihan HE (1997). Medicine and the Dublin Philosophical Society (1683–1686). Ir J Med Sci.

